# Combined Obstetric and Thrombotic Complications Are Associated with Features Suggestive of an Immune-Enriched Phenotype Among Women with Thrombotic Antiphospholipid Syndrome (APS)

**DOI:** 10.3390/diagnostics16121794

**Published:** 2026-06-10

**Authors:** Paula David, Gabrielle M. Santos, Or Hen, Juan Luis Ontiveros-Austria, Rotem Liran, Sergio Rodriguez-Rodriguez, Yonatan Shneor Patt, Erica Okazaki, Marina Colella, Gabriela G. Yamaguti-Hayakawa, Shada Azem, Yoav Elizur, Joyce Annichino-Bizzacchi, Erich V. De Paula, Shaked Beladev, Ophira Salomon, Roberta Demichelis-Gomez, Ana Barrera-Vargas, Howard Amital, Yehuda Shoenfeld, Paula Villaça, Fernanda A. Orsi, Omer Gendelman

**Affiliations:** 1 Rheumatology Unit, Sheba Medical Center-Tel Hashomer, Ramat Gan 5262100, Israel; paulardavid@gmail.com (P.D.); shatha.azem@hotmail.com (S.A.); 2 Leeds Institute of Rheumatic and Musculoskeletal Medicine, University of Leeds, Leeds LS1 3EX, UK; orhen92@gmail.com; 3 Discipline of Hematology, Hemotherapy and Cell Therapy, Department of Internal Medicine, University of São Paulo Medical School, São Paulo 05403-000, Brazil; gabi.msantos93@gmail.com (G.M.S.); erica.okazaki@hc.fm.usp.br (E.O.); paula.villaca@hc.fm.usp.br (P.V.); 4 State Institute of Hematology Arthur de Siqueira Cavalcanti (HEMORIO), Rio de Janeiro 20211-030, Brazil; 5 Internal Medicine C, Sheba Medical Center-Tel Hashomer, Ramat Gan 5262100, Israel; 6 Department of Hematology and Oncology, Instituto Nacional de Ciencias Médicas y Nutrición Salvador Zubirán, Mexico City 14080, Mexico; juanlontiverosa@gmail.com (J.L.O.-A.); sergio.rodriguez.rodriguez@gmail.com (S.R.-R.); robertademichelis@gmail.com (R.D.-G.); 7 Department of Internal Medicine E, Meir Medical Center, Kfar Saba 4428164, Israel; rotemli777@gmail.com (R.L.); shakedbel94@gmail.com (S.B.); 8 Internal Medicine B, Sheba Medical Center-Tel Hashomer, Ramat Gan 5262100, Israel; yopatt123@gmail.com (Y.S.P.); yj.elizur@gmail.com (Y.E.); howard.amital@sheba.health.gov.il (H.A.); 9 Hematology and Hemotherapy Center, Faculty of Medical Sciences, University of Campinas (UNICAMP), Campinas 13083-887, Brazil; marinasp@unicamp.br (M.C.); gabrielayamaguti@gmail.com (G.G.Y.-H.); joyce@unicamp.br (J.A.-B.); erich@unicamp.br (E.V.D.P.); ferorsi@unicamp.br (F.A.O.); 10 School of Medical Sciences, University of Campinas (UNICAMP), Campinas 13083-887, Brazil; 11 Thrombosis Unit, Sheba Medical Center-Tel Hashomer, Ramat Gan 5262100, Israel; ophira.salomon@sheba.health.gov.il; 12 Department of Rheumatology, Instituto Nacional de Ciencias Médicas y Nutrición Salvador Zubirán, Mexico City 14080, Mexico; ana.barrerav@incmnsz.mx; 13 Zabludowicz Center for Autoimmune Diseases, Sheba Medical Center-Tel Hashomer, Ramat Gan 5262100, Israel; yehuda.shoenfeld@sheba.health.gov.il; 14 Dina Recanati School of Medicine, Reichman University, Herzliya 4610101, Israel

**Keywords:** antiphospholipid syndrome, pregnancy complications, hematologic, thrombosis, antinuclear antibodies, thrombocytopenia, autoimmune diseases

## Abstract

**Background/Objectives**: To determine whether obstetric antiphospholipid syndrome (oAPS) among women with primary thrombotic APS (tAPS) defines a distinct immunologic phenotype or influences thrombotic characteristics and recurrence risk. **Methods**: This predefined subgroup analysis was conducted within a multicenter retrospective cohort study of 663 patients with persistent antiphospholipid antibody (aPL) positivity and objectively confirmed thrombosis. The present analysis included 295 women with primary tAPS and a history of pregnancy. Participants were classified as oAPS or non-oAPS according to the revised Sydney criteria. Demographic, clinical, obstetric, immunologic, and hematologic features at baseline were compared. Univariable and multivariable logistic regression analyses were performed to identify predictors of oAPS and determinants of arterial versus venous thrombosis. **Results**: Of 295 women, 115 (39.0%) met oAPS criteria. ANA positivity (33.0% vs. 19.0%, *p* = 0.008), triple aPL positivity (46.0% vs. 29.1%, *p* = 0.003), and severe thrombocytopenia (22.8% vs. 13.3%, *p* = 0.035) were more frequent in oAPS. In multivariable analysis, ANA positivity remained independently associated with oAPS (adjusted OR 1.94, 95% CI 1.05–3.56; *p* = 0.033), whereas triple aPL positivity and thrombocytopenia did not remain independently significant after adjustment. Thrombotic phenotype and recurrence rates were similar between oAPS and non-oAPS groups. Obstetric history was not associated with arterial events; hypertension was the strongest predictor of arterial thrombosis (adjusted OR 2.61, *p* < 0.001). **Conclusions**: In primary tAPS, oAPS is associated with features suggestive of an immune-enriched phenotype, particularly ANA positivity, without evidence of a distinct thrombotic phenotype or increased recurrence risk. Integration of obstetric and immunologic features may help guide risk stratification and hypothesis generation for future studies.

## 1. Introduction

Women with primary thrombotic antiphospholipid syndrome (tAPS) have a substantially higher risk of recurrent thrombosis than women with isolated obstetric APS (oAPS), independent of the antiphospholipid antibody (aPL) profile [1,2]. A prospective cohort study reported markedly higher recurrence rates in tAPS, with recurrence reaching up to 30–35% within 5 years, compared with rates of approximately 2–5% in isolated oAPS [1].

These observations suggest that the syndrome-defining clinical phenotype (thrombotic versus obstetric) captures prognostic information beyond antibody profile alone. The risk of arterial thrombosis in APS is strongly associated with high-risk aPL profiles, particularly lupus anticoagulant (LA) positivity and triple antibody positivity, as well as with traditional cardiovascular risk factors such as hyperlipidemia and reduced renal function [3,4]. However, antibody profiles alone do not fully explain the heterogeneity in vascular bed involvement or the distinction between arterial and venous events [3,4]. Women whose APS initially manifests with pregnancy morbidity generally exhibit a lower long-term thrombotic risk than those with tAPS, although this risk is not negligible and appears to be modulated by both serological and clinical factors [1,5].

Cohort studies evaluating women with oAPS or aPL carriers without prior thrombosis have shown that the incidence of a first thrombotic event in isolated oAPS is comparable to that observed in asymptomatic aPL carriers, but that when thrombosis occurs, arterial events predominate [6,7]. Across studies, LA remains the most consistent predictor of thrombosis in oAPS and in aPL carriers without pregnancy morbidity [7].

Importantly, obstetric complications do not seem to modify the risk of thrombotic recurrence once thrombosis has occurred [3,7,8]. While long-term follow-up studies indicate that a minority of women with isolated oAPS may eventually develop thrombotic events—reported rates range from 20–35% over extended follow-up—recurrence rates remain substantially lower than those observed in tAPS, with risk concentrated in patients with high-risk antibody profiles or additional cardiovascular risk factors [1,9].

Accordingly, current consensus guidelines do not support long-term antithrombotic therapy for women with isolated oAPS in the absence of additional risk factors, although close monitoring is recommended for those with LA positivity, triple aPL positivity, or coexisting cardiovascular risk factors [5,7,8]. Beyond aPL profile, attention has turned to broader immunologic markers, including antinuclear antibodies (ANAs).

ANAs are frequently detected in patients with APS without systemic autoimmune disease, yet their clinical significance remains uncertain. ANAs positivity may reflect broader immune activation and loss of immune tolerance, even in the absence of overt connective tissue disease. Recent studies suggest that ANA-positive APS patients represent a distinct immune-enriched phenotype characterized by higher frequencies of triple aPL positivity, complement abnormalities, hematologic manifestations, and other non-criteria autoimmune features [10]. Prior studies assessing ANAs in APS have yielded inconsistent findings, largely due to heterogeneous populations, inclusion of secondary APS and systemic lupus erythematosus, and limited stratification by clinical phenotype [11,12,13].

Available evidence suggests that women with isolated oAPS form a distinct risk subgroup, with lower overall thrombotic risk than tAPS but a meaningful burden of arterial events in the presence of high-risk serologic or clinical features. However, whether oAPS within established primary tAPS is associated with an immune-enriched phenotype or alters thrombotic phenotype and recurrence risk remains unclear; therefore, we evaluated predictors of obstetric complications among women with primary tAPS and examined associations of oAPS with thrombotic phenotype, recurrence, and immunologic features.

## 2. Materials and Methods

### 2.1. Study Design and Source Cohort

This study is a predefined subgroup analysis of a previously described multicenter retrospective cohort of patients with primary tAPS [14]. The source population initially included 1377 patients with APS recruited across five tertiary referral centers: Sheba Medical Center and Meir Medical Center (Israel), the University of São Paulo (USP) and the University of Campinas (UNICAMP) (Brazil), and Instituto Nacional de Ciencias Médicas y Nutrición Salvador Zubirán (INCMNSZ) (Mexico).

Patients with secondary APS or established systemic autoimmune rheumatic diseases were excluded from the parent cohort. Excluded diseases included systemic lupus erythematosus, Sjögren’s syndrome, rheumatoid arthritis, systemic sclerosis, idiopathic inflammatory myopathies, systemic vasculitis, mixed connective tissue disease, and other defined connective tissue diseases. Secondary autoimmune diseases were identified based on at least one of the following: (a) a documented physician-confirmed diagnosis in the medical record; (b) concordant ICD coding entered by at least two physicians; or (c) fulfillment of the most updated applicable ACR/EULAR classification criteria based on retrospective review of available clinical and laboratory data in patients without a previously established autoimmune diagnosis [15,16,17,18,19]. A total of 294 patients were therefore classified as secondary APS and excluded.

Additional predefined exclusion criteria included non-thrombotic APS (*n* = 32), failure to meet revised Sydney APS classification criteria (*n* = 140), insufficient or unavailable primary data (*n* = 193), active malignancy at diagnosis (*n* = 7), and age < 18 years (*n* = 48). The final parent cohort comprised 663 patients with primary thrombotic APS and persistent aPL positivity [14]. Thrombotic events were confirmed by imaging or biopsy when applicable.

### 2.2. Patient Selection for the Current Analysis

From the original cohort of 663 patients, we restricted the current study population to women with a documented history of at least one pregnancy, yielding 295 women eligible for inclusion.

All included patients had persistent aPL positivity confirmed at least 12 weeks apart and a history of tAPS. Patients with isolated oAPS without a history of thrombosis were excluded from the parental study design.

### 2.3. Definition of Obstetric APS Phenotype

Women were classified according to the presence or absence of oAPS based on the revised Sydney (Sapporo) classification criteria. The oAPS phenotype was defined by the presence of at least one of the following clinical manifestations in the setting of persistent aPL positivity: 1. One or more unexplained fetal deaths at or beyond the 10th gestational week; 2. One or more premature births before 34 weeks’ gestation due to placental insufficiency, preeclampsia, or eclampsia; or 3. Three or more unexplained consecutive spontaneous abortions before the 10th gestational week.

Women with a pregnancy history who did not meet any obstetric criteria were classified as non-oAPS.

### 2.4. Data Collection and Laboratory Assessment

Demographic, clinical, obstetric, and laboratory data were extracted retrospectively from electronic medical records. Laboratory data were derived from documented clinical testing performed as part of routine care.

aPL testing (lupus anticoagulant [LA], anticardiolipin [aCL], and anti-β2 glycoprotein I [anti-β2GPI] antibodies) was performed locally in specialized laboratories at each participating center according to internationally accepted laboratory standards [20]. Persistent antibody positivity was verified by repeat measurements obtained at least 12 weeks after the initial positive test, in accordance with revised APS classification criteria. Because several coagulation-related assays may be affected by acute thrombosis and anticoagulant treatment, laboratory results were interpreted within the clinical context, and repeat testing under more reliable conditions was performed when deemed necessary by the treating thrombosis/hemostasis specialists.

For the purposes of the present study, laboratory parameters included in the analysis-including immunologic, hematologic, and biochemical variables—were collected within 12 months of the index thrombotic event. When multiple measurements were available during this period, values closest to the thrombotic presentation were preferentially selected.

Detailed laboratory methodologies for aPL testing and other assays are described in the parent cohort study [14]. Triple aPL positivity was defined as concurrent positivity for LA, aCL, and anti-β2GPI.

Complement measurements were not systematically available for all patients because testing was performed according to routine clinical practice and physician discretion rather than a predefined study protocol. Given the clinically guided nature of complement testing, missingness was considered likely not completely at random; therefore, data imputation was not performed.

Cardiometabolic comorbidities assessed included hypertension, diabetes mellitus, dyslipidemia, obesity, smoking status, and chronic kidney disease (eGFR < 60 mL/min/1.73 m^2^), using standardized definitions consistent with the parent cohort [14].

### 2.5. Thrombotic Events

Thrombotic events were categorized as arterial, venous, or combined based on vascular territory and imaging confirmation. Recurrent thrombosis was defined as a new objectively confirmed thrombotic event occurring after the index thrombosis, regardless of vascular bed.

### 2.6. Statistical Analysis

Baseline demographic characteristics, cardiovascular risk factors, APS-related clinical features, and aPL profiles were compared between women with and without an oAPS phenotype. LA, aCL, and anti-β2GPI antibodies were analyzed according to isotype, and triple positivity was defined as positivity for all three aPL tests. Continuous variables are presented as median with interquartile range (IQR) and were compared using the Mann–Whitney U test. Categorical variables are presented as counts and percentages and were compared using the χ^2^ test or Fisher’s exact test, as appropriate.

The comparisons presented in Table 1 were primarily intended as descriptive and exploratory analyses aimed at characterizing baseline clinical, serologic, and immunologic differences between groups rather than as formal hypothesis-testing primary outcomes. Accordingly, and given the exploratory nature of the study, no formal correction for multiple testing was applied. All *p*-values are therefore presented as nominal values to allow a comprehensive interpretation of potential clinical and biologic patterns within the cohort.

To identify factors independently associated with the oAPS phenotype, univariable logistic regression models were first performed, with oAPS as the dependent variable. Variables considered clinically relevant were then entered into a multivariable logistic regression model using the enter method, including age at first thrombosis, ANA positivity, thrombocytopenia < 100 × 10^3^/μL, hypertension, diabetes mellitus, dyslipidemia, and obesity (BMI ≥ 30 kg/m^2^).

A separate set of univariable and multivariable logistic regression analyses was performed to evaluate predictors of arterial versus venous first thrombotic event, with arterial thrombosis coded as 1 and venous thrombosis as 0. Variables included in the multivariable model were selected based on clinical relevance and <15% missing data and comprised oAPS phenotype, age at first thrombosis, diabetes mellitus, hypertension, dyslipidemia, obesity, and severe thrombocytopenia (<100 × 10^3^/µL) and lupus anticoagulant positivity. Smoking status, ANA positivity, and complement levels were excluded from this model due to substantial missingness.

Odds ratios (ORs) are presented with 95% confidence intervals (CIs). Statistical significance was defined as a two-sided *p* value < 0.05. All analyses were performed using IBM SPSS Statistics, version 31.0.1.0 (IBM Corp., Armonk, NY, USA). Two-sided *p* values < 0.05 were considered statistically significant.

## 3. Results

### 3.1. Study Population

Among the 663 patients included in the parent cohort of thrombotic primary APS, 390 (59.0%) were women. Of these, 295 women (75.6%) had a documented history of at least one pregnancy and constituted the study population for the present analysis. All included women had persistent aPL positivity and a history of objectively confirmed arterial and/or venous thrombosis.

Within this population, 115 women (39.0%) fulfilled the revised Sydney criteria for oAPS, while 180 women (61.0%) did not meet obstetric criteria (non-oAPS). Among women with oAPS, the distribution of obstetric clinical criteria is shown in Table 1. Patients with isolated oAPS without thrombosis were not included by design.

### 3.2. Baseline Characteristics and Antiphospholipid Antibody Profile

Baseline characteristics and aPL profiles according to obstetric phenotype are summarized in Table 1.

The prevalence of lupus anticoagulant was high in both groups (82.2% in non-oAPS vs. 87.8% in oAPS; *p* = 0.196). Frequencies of anticardiolipin and anti-β2-glycoprotein I antibodies, stratified by isotype, were numerically higher among oAPS patients but did not differ significantly between groups.

Triple aPL positivity was significantly more frequent in the oAPS group compared with the non-oAPS group (46.0% vs. 29.1%, *p* = 0.003). In univariate analysis, triple aPL positivity was associated with approximately twofold higher odds of having an oAPS phenotype (OR 2.08, 95% CI 1.29–3.37, *p* = 0.003). However, after multivariable adjustment, this association was attenuated and no longer statistically significant (adjusted OR 1.51, 95% CI 0.87–2.64, *p* = 0.145).

Cardiometabolic comorbidities differed between groups. Hypertension was significantly more prevalent among non-oAPS patients (50.0% vs. 33.9%, *p* = 0.007). Smoking status also differed significantly between groups (*p* = 0.037), whereas rates of diabetes mellitus, dyslipidemia, obesity, and chronic kidney disease were comparable.

Immunologic and hematologic parameters differed modestly between groups. ANAs positivity was significantly more frequent in oAPS compared with non-oAPS (33.0% vs. 19.0%, *p* = 0.008). Severe thrombocytopenia was also more prevalent in oAPS (22.8% vs. 13.3%, *p* = 0.035), although this association did not remain statistically significant after multivariable adjustment (adjusted OR 1.69, 95% CI 0.80–3.59; *p* = 0.172).

Low C3 (<90 mg/dL) was numerically more frequent in oAPS than in non-oAPS, although the between-group difference did not reach statistical significance (14.9% vs. 7.5%, *p* = 0.078). Low C4 (<10 mg/dL) was similar between groups (3.6% vs. 5.7%, *p* = 0.485). A missingness analysis comparing patients with available versus unavailable complement measurements is presented in Supplementary Table S1 and demonstrates no major differences in baseline thrombotic or cardiometabolic characteristics between groups. The main difference observed was a higher prevalence of thrombocytopenia among patients who underwent complement testing, suggesting that, in routine clinical practice, physicians may preferentially assess complement consumption in APS patients presenting with hematologic abnormalities suggestive of immune dysregulation.

Among ANA-positive patients with available pattern data (*n* = 57 of 67 ANA+ patients), the most frequent ANA pattern group was nuclear homogeneous (15/57, 26.3%), followed by nuclear speckled (non-DFS) (14/57, 24.6%), cytoplasmic/cytoskeletal patterns (10/57, 17.5%), DFS70-like dense fine speckled patterns (9/57, 15.8%), and nucleolar patterns (9/57, 15.8%). When stratified by oAPS phenotype, no significant differences were observed in the distribution of ANA pattern groups between patients with oAPS and those without obstetric manifestations (Pearson χ^2^ = 3.58, df = 4, *p* = 0.466). Nuclear homogeneous and nuclear speckled patterns were similarly represented in both groups, and DFS70-like patterns were not enriched in either phenotype.

In an exploratory analysis of 57 ANA-positive patients with available titer data, the median ANA titer was 1:160 (IQR 80–320) in both the oAPS and non-oAPS groups (*p* = 0.326). While these findings suggest a similar intensity of ANA expression across phenotypes, this sub-analysis was likely underpowered to detect subtle differences.

### 3.3. Obstetric Manifestations

Among the 115 women fulfilling oAPS criteria, the most frequent clinical manifestations were late fetal loss (52.2%) and placental APS morbidity, including preeclampsia, eclampsia, or placental insufficiency leading to delivery before 34 weeks of gestation (53.9%). Recurrent early pregnancy loss occurred in 16.5% of cases (Table 2).

### 3.4. Obstetric APS Phenotype and Factors Associated

In univariable logistic regression analyses, ANA positivity was associated with increased odds of an oAPS phenotype (OR 2.02, 95% CI 1.13–3.62, *p* = 0.018), while hypertension was inversely associated with oAPS (OR 0.51, 95% CI 0.32–0.83, *p* = 0.007). Thrombocytopenia showed a borderline association with oAPS but did not reach statistical significance (OR 2.01, 95% CI 0.99–4.07; *p* = 0.053). Triple aPL positivity was also associated with increased odds of oAPS in univariable analysis (OR 2.08, 95% CI 1.29–3.37; *p* = 0.003). Other variables, including age at first thrombosis, hypertension, diabetes mellitus, dyslipidemia, and obesity, were not independently associated with oAPS.

In multivariable logistic regression analysis, only ANA positivity remained independently associated with the oAPS phenotype (adjusted OR 1.94, 95% CI 1.05–3.56; *p* = 0.033) (Table 3).

Thrombotic features according to obstetric phenotype are shown in Table 1. Women with oAPS experienced their first thrombotic event at a significantly younger age compared with non-oAPS patients (median 31.9 vs. 40.4 years; *p* = 0.003). This age difference remained consistent in direction after multivariable adjustment, although it did not reach statistical significance in the adjusted model (adjusted OR per year 0.98, 95% CI 0.97–1.00; *p* = 0.091).

The distribution of arterial versus venous first thrombosis did not differ between groups (*p* = 0.789), nor did the median number of thrombotic events (*p* = 0.360). The proportion of patients experiencing recurrent thrombosis was similar between oAPS and non-oAPS groups (40.9% vs. 45.6%; *p* = 0.430). Among patients with recurrent thrombosis, the distribution of recurrent arterial and venous events was similar between oAPS and non-oAPS patients. Recurrent venous events occurred in 20/45 (44.4%) women with oAPS versus 46/81 (56.8%) without oAPS, while recurrent arterial events occurred in 24/45 (53.3%) versus 35/81 (43.2%), respectively. Combined arterial and venous recurrence was rare.

In analyses evaluating determinants of thrombotic phenotype, the oAPS phenotype was not associated with the type of first thrombotic event in either univariable or multivariable models. After adjustment, oAPS was not independently associated with arterial versus venous thrombosis (adjusted OR 1.25, 95% CI 0.72–2.18, *p* = 0.427).

In contrast, hypertension was strongly and independently associated with arterial first thrombosis (adjusted OR 2.61, 95% CI 1.46–4.67, *p* < 0.001). Obesity was independently associated with lower odds of arterial thrombosis (adjusted OR 0.47, 95% CI 0.26–0.85, *p* = 0.013). Age at first thrombosis, diabetes mellitus, dyslipidemia, severe thrombocytopenia, and lupus anticoagulant positivity were not independently associated with thrombotic phenotype after multivariable adjustment (Table 4).

## 4. Discussion

In this large multicenter cohort of women with primary thrombotic APS, the coexistence of obstetric APS manifestations was associated with higher frequencies of ANA positivity and triple aPL positivity. Women with oAPS were also younger at first thrombosis and demonstrated a higher prevalence of severe thrombocytopenia in unadjusted analyses, although these associations did not remain independently significant after multivariable adjustment. Importantly, thrombotic phenotype and recurrence rates were comparable between oAPS and non-oAPS groups, suggesting that the coexistence of obstetric manifestations may reflect greater immune dysregulation rather than a more severe thrombotic phenotype.

Most prior comparative studies have focused on isolated oAPS versus isolated thrombotic APS, consistently reporting higher thrombotic recurrence rates and a greater burden of hematologic abnormalities in tAPS [1,2]. Interpretation of immunologic markers—particularly ANA—in these studies has often been complicated by population heterogeneity, including the inclusion of secondary APS and systemic autoimmune diseases such as systemic lupus erythematosus, in which ANA positivity reflects underlying disease rather than APS-specific immune activation.

Recent data from the APS ACTION registry addressed this limitation by restricting analyses to aPL-positive patients without concomitant systemic autoimmune rheumatic diseases and demonstrated that ANA positivity identifies a more immune-enriched phenotype characterized by hematologic abnormalities and complement consumption, but it is not independently associated with thrombosis or pregnancy morbidity. On the other hand, ANA-negative female patients presented with a higher number of pregnancies (*p* = 0.018) and number of live births (*p* = 0.014) [10].

A key strength of this study is the exclusive inclusion of women with primary tAPS, with systematic exclusion of secondary APS and defined systemic autoimmune diseases, enabling focused assessment of a clinically homogeneous, high-risk subgroup. Within this population, ANA positivity was significantly more frequent in women with concomitant oAPS, indicating that ANA reflects an immune-enriched phenotype that tends to manifest as obstetric involvement rather than influencing thrombotic recurrence. The higher prevalence of triple aPL positivity and the numerically increased prevalence of severe thrombocytopenia among women with combined obstetric and thrombotic manifestations further support the presence of a more immune-enriched phenotype, although the thrombocytopenia association did not remain independently significant after multivariable adjustment.

Our prior cluster analyses of primary tAPS identified a reproducible immune-enriched cluster characterized by younger age, ANA positivity, thrombocytopenia, complement abnormalities, and a lower burden of classic cardiovascular comorbidities such as hypertension [14]. The similarity between this cluster and the oAPS subgroup in the present study suggests that obstetric morbidity overlays an existing immunologic trait rather than altering the thrombotic trajectory. Importantly, pregnancy-related variables were not included in the clustering analysis, indicating that this immune-enriched phenotype emerged independently of obstetric history.

Recent studies in venous thromboembolism populations further support the broader concept that readily available laboratory parameters may help identify biologically and clinically distinct thrombotic phenotypes. In the RIETE registry [21,22], baseline LDL cholesterol and hemoglobin levels were associated with differential bleeding risk and clinical outcomes during anticoagulation, suggesting that simple laboratory biomarkers may provide incremental prognostic information beyond conventional thrombotic classification. Although these studies were conducted in VTE rather than APS, they reinforce the paradigm that routinely available laboratory variables may reflect underlying biological heterogeneity within thrombotic disorders. In this context, our findings support ANA positivity and potentially triple positivity and thrombocytopenia as markers of an immune-enriched APS phenotype.

Despite immune enrichment, oAPS was not independently associated with thrombotic phenotype or recurrence after multivariable adjustment. Prior cohorts of women with isolated oAPS show that they are not protected from thrombosis and may experience relatively frequent arterial, particularly cerebrovascular, events over long-term follow-up [3,4]. These risks are driven mainly by the presence and persistence of lupus anticoagulant rather than obstetric morbidity itself, highlighting the central role of pathogenic aPL [3,4]. In our cohort of women with established primary tAPS, lupus anticoagulant prevalence was similarly high in those with and without oAPS, which likely explains the comparable arterial-venous distribution and recurrence rates. Given previous reports of arterial-predominant thrombosis in oAPS, we specifically evaluated predictors of arterial versus venous thrombosis in multivariable models (Table 4) to determine whether obstetric history contributed independently beyond LA status and vascular risk factors. These analyses reinforce that once thrombosis has occurred, traditional vascular risk factors dominate subsequent thrombotic behavior rather than obstetric history. Consistent with prior studies, hypertension emerged as the strongest independent predictor of arterial thrombosis [3,23], while obesity was related to more venous events, as previously established [24,25,26,27]. This pattern is biologically plausible, as obesity is known to promote venous thromboembolism through chronic low-grade inflammation, endothelial dysfunction, increased fibrinogen and plasminogen activator inhibitor-1 levels, impaired fibrinolysis, and venous stasis [27,28,29,30]. These mechanisms may synergize with the prothrombotic effects of antiphospholipid antibodies, preferentially amplifying venous rather than arterial thrombosis. The concordance of our findings with prior APS cohorts supports the biological plausibility and internal consistency of our multivariable models and further emphasizes the importance of aggressive management of modifiable cardiovascular and metabolic risk factors in APS patients.

Importantly, the association between ANA positivity and obstetric morbidity is not unique to APS, suggesting that ANA may function as a broader marker of immune dysregulation relevant to pregnancy outcomes. In a prospective study of women with positive aPL and prior adverse pregnancy outcomes (predominantly non-criteria oAPS), an ANA titer ≥ 1:160 independently predicted adverse pregnancy outcomes, while lower first-trimester C3 levels were associated with fetal loss, supporting an immune- and complement-mediated contribution to placental injury [31]. Two independent meta-analyses of case–control studies evaluating women with recurrent miscarriage or recurrent pregnancy loss found significantly higher ANA positivity in affected women compared with healthy controls, supporting ANA as a risk factor [32,33]. Our findings extend this by showing that, in women with established primary tAPS, ANA positivity preferentially associates with obstetric manifestations without increasing thrombotic recurrence.

From a clinical perspective, identification of an immune-enriched phenotype characterized by ANA positivity, triple aPL positivity, and possibly hematologic abnormalities may help identify women who warrant closer monitoring during pregnancy, particularly in the setting of prior obstetric morbidity or additional immune features. Such patients may potentially benefit from a more individualized multidisciplinary approach integrating thrombosis, rheumatology, and maternal-fetal medicine expertise. Although prospective validation is needed, these findings further support ongoing interest in adjunctive immunomodulatory strategies in selected high-risk obstetric APS patients.

The identification of an immune-enriched APS phenotype has potential therapeutic implications, while long-term anticoagulation remains the cornerstone of tAPS management [4]. Adjunctive immunomodulatory strategies may be relevant in selected subgroups, particularly in the obstetric setting. Hydroxychloroquine (HCQ) is widely used in rheumatic diseases for its immunomodulatory and anti-inflammatory effects, and has been proposed as an adjunctive therapy in APS based on observational and preclinical evidence suggesting potential antithrombotic and placental protective effects [34,35]. In APS, HCQ has complex pleiotropic effects on endothelial cells, platelets, and immune pathways that offer a biologic rationale for adjunctive use in both thrombotic and obstetric manifestations [35].

Over the past decade, a significant shift in understanding obstetric APS has taken place. What was once viewed primarily as a thrombotic disorder is now recognized as a condition in which immune-mediated, complement-driven inflammation plays a central role. While complement-related findings in our cohort were exploratory and did not reach statistical significance, prior mechanistic and translational studies have increasingly implicated complement activation in the pathogenesis of obstetric APS. Experimental and translational studies suggest that antiphospholipid antibodies can initiate complement activation, a process supported by findings of placental complement deposition and by clinical associations between hypocomplementemia and adverse pregnancy outcomes [31,36,37,38]. In the PROMISSE study, women with poor outcomes showed substantially higher levels of complement activation fragments [37]. Experimental models reinforce this mechanism: exposure to antiphospholipid antibodies results in complement activation, recruitment of neutrophils, and release of inflammatory mediators such as TNF-α, culminating in placental injury; notably, fetal loss is prevented in C5-deficient mice or with C5 blockade [36,37,38,39]. Collectively, these findings support a model in which complement activation, neutrophil-mediated inflammation, and cytokine release—rather than thrombosis alone—drive placental dysfunction in oAPS, providing a biological rationale for targeting inflammatory pathways in selected high-risk pregnancies [40,41].

In pregnancy, retrospective and observational data indicate that HCQ added to standard therapy (low-dose aspirin plus low-molecular-weight heparin) is associated with higher live-birth rates and lower rates of obstetric complications in women with oAPS compared with standard therapy alone [35,42]. Systematic reviews and meta-analyses of observational studies support a beneficial association of HCQ with improved pregnancy outcomes, although randomized controlled trial data are not yet available, and study populations remain heterogeneous.

The absence of increased thrombotic recurrence among women with oAPS in our cohort suggests that immune enrichment per se is insufficient to confer excess thrombotic risk once APS-related thrombosis has occurred. This observation aligns with current guidelines that prioritize antiphospholipid antibody profile and traditional cardiovascular risk factors over obstetric phenotype for long-term thrombotic risk stratification in APS [4,43]. Nevertheless, in the pregnancy context, immune enrichment—manifested by ANA positivity and triple aPL positivity and potentially accompanied by hematologic or complement abnormalities in selected patients—may help identify APS subgroups in whom HCQ could be considered as adjunctive therapy, given its emerging association with improved obstetric outcomes and established safety profile in pregnancy [35,42].

Emerging data suggest that targeted immunomodulation beyond HCQ may benefit selected women with refractory oAPS. Small observational studies—and more recently a phase 2 study of anti-TNF therapy—have reported improved pregnancy outcomes in women who failed standard aspirin and LMWH treatment [44,45]. Although evidence remains limited and non-randomized, these findings support a contribution of immune-mediated mechanisms to obstetric morbidity and align with the immune-enriched phenotype observed in our cohort. Immunologic profiling may help identify patients who could benefit from such approaches, but anti-TNF therapy remains experimental and should be reserved for highly selected refractory cases in specialized centers.

Several limitations should be acknowledged. First, the retrospective design precludes assessment of temporal relationships between obstetric morbidity and thrombosis. Accordingly, we cannot determine whether obstetric complications preceded or followed thrombotic events. In addition, the older age at first thrombosis observed in the non-oAPS group—often beyond typical reproductive years—raises the possibility that some women may not have been diagnosed with APS during pregnancy and were therefore classified as non-oAPS due to absence of documented obstetric manifestations. However, age at thrombosis was not independently associated with obstetric manifestations in multivariable analysis, suggesting that ascertainment bias alone is unlikely to fully explain the observed differences. The sample size of our study was determined by the total number of eligible patients meeting strict inclusion criteria across multiple specialized tertiary centers, representing one of the larger available cohorts of primary thrombotic APS. Nevertheless, the study may still have been underpowered to detect modest independent associations for some immunologic variables after multivariable adjustment. Complement levels and ANAs patterns were unavailable for a subset of patients, since they are not routinely collected for primary APS, and they were obtained according to physician discretion in real-world clinical practice. Consequently, complement testing was likely influenced by clinical suspicion of immune dysregulation, introducing potential selection bias. However, complement variables were included only as exploratory descriptive parameters and were not incorporated into the primary regression outcome models. Findings regarding complement should therefore be interpreted as exploratory and hypothesis-generating, warranting further validation in larger prospective cohorts and international registries.

The multivariable model demonstrated modest explanatory performance, likely reflecting both the complex and multifactorial nature of APS and the retrospective multicenter design, with incomplete availability of potentially relevant variables such as detailed obstetric history, treatment exposure, and non-criteria autoantibodies. However, the primary objective of the model was not to develop a predictive tool but rather to identify independent clinical and immunologic associations with the oAPS phenotype. Additionally, treatment exposure—including hydroxychloroquine use—was not uniformly captured. Finally, although patients with documented systemic autoimmune rheumatic diseases were systematically excluded using predefined classification-based criteria, the retrospective design does not allow complete exclusion of undiagnosed, evolving, or subclinical autoimmune disease. Nevertheless, this study is strengthened by its multicenter design, rigorous restriction to thrombotic APS, systematic exclusion of secondary autoimmune disease, and concordance with independent phenotype-driven clustering analyses.

## 5. Conclusions

In conclusion, among women with primary thrombotic antiphospholipid syndrome, the concomitant presence of oAPS is associated with features suggestive of an immune-enriched phenotype, particularly ANA positivity, with additional hematologic and immunologic features suggestive of immune dysregulation, including thrombocytopenia. Importantly, this phenotype was not associated with a distinct thrombotic presentation or increased recurrence risk. Integration of obstetric history with immunologic profiling—supported by phenotype-driven analyses—may help identify APS subgroups in whom adjunctive immunomodulatory strategies such as hydroxychloroquine warrant consideration during pregnancy to mitigate obstetric complications and support a more personalized approach to APS management.

## Figures and Tables

**Table 1:** Baseline characteristics and antiphospholipid antibody profile of the study population, stratified by obstetric APS phenotype.

Characteristic	Non-Obstetric APS (*N* = 180)	Obstetric APS (*N* = 115)	*p* Value
Age at first thrombosis, in years—median (IQR)	40.4 (26.6–57)	31.9 (25–47)	0.003
Lupus anticoagulant, *n* (%)	148 (82.2)	101 (87.8)	0.196
aCL positivity (any), *n* (%)	33 (18.3)	31 (27)	0.080
aCL positivity (IgM), *n* (%)	23 (12.8)	24 (20.9)	0.069
aCL positivity (IgG), *n* (%)	11 (6.5)	10 (9.5)	0.354
Anti-β2GPI positivity (any), *n* (%)	51 (28.6)	40 (39.6)	0.215
Anti-β2GPI positivity (IgM), *n* (%)	41 (26.5)	31 (31.3)	0.402
Anti-β2GPI positivity (IgG), *n* (%)	17 (11.4)	15 (16.9)	0.234
Triple positivity, *n* (%)	52 (29.1)	52 (46)	0.004
Hypertension, *n* (%)	90 (50)	39 (33.9)	0.007
Diabetes mellitus, *n* (%)	35 (20)	15 (13.3)	0.140
Hyperlipidemia, *n* (%)	122 (67.8)	65 (57)	0.060
Obesity, *n* (%)	69 (39.7)	35 (32.7)	0.240
Smoking status, *n* (%)			0.037
Never	129 (75.4)	82 (79.6)	
Active	36 (21.1)	12 (11.7)	
Past	6 (3.6)	9 (8.7)	
CKD/eGFR < 60, *n* (%)	20 (11.1)	12 (11.7)	0240
ANA positivity, *n* (%)	32 (19) *	37(33) *	0.008
Low C3, *n* (%)	10 (7.5) **	14 (12.2)	0.078
Low C4, *n* (%)	7 (5.7) ***	3 (2.6) ***	0.585
Thrombocytopenia < 100 × 10^3^/μL	24 (13.3)	26 (22.8)	0.035
Type of first thrombosis, *n* (%)			0.789
Venous	107 (59.4)	69 (60.0)	
Arterial	69 (38.3)	42 (36.5)	
Both arterial and venous	4 (2.2)	4 (3.5)	
Recurrent thrombosis, *n* (%)	82 (45.6)	47 (40.9)	0.430

* ANAs results were available for 168/180 non-oAPS patients and 112/115 oAPS patients; ** C3 values were available on 133/180 non-oAPS patients and 87/115 oAPS patients; *** C4 was available on 123/180 non-oAPS patients and 84/115 oAPS patients.

**Table 2:** Obstetric antiphospholipid syndrome (APS) clinical criteria among women with obstetric APS phenotype (*N* = 115).

Obstetric APS Clinical Criterion	*n* (%)
Recurrent early pregnancy loss (≥3 consecutive spontaneous abortions before the 10th gestational week, not due to maternal anatomical/hormonal maternal causes and parental chromosomal causes)	19/115 (16.5)
Late fetal loss (≥1 unexplained fetal death of a morphologically normal fetus at or beyond the 10th gestational week)	60/115 (52.2)
Placental APS morbidity (≥1 premature birth of a morphologically normal neonate before 34 weeks due to severe pre-eclampsia, eclampsia, or placental insufficiency)	62/115 (53.9)

**Table 3:** Multivariable logistic regression identifying clinical and immunologic features associated with an obstetric APS phenotype.

Variable	Crude OR (95% CI)	*p* Value	Adjusted OR (95% CI)	*p* Value
Age during first thrombosis (per year)	0.98 (0.97–1.00)	0.09	0.99 (0.97–1.01)	0.153
ANA positivity	2.02 (1.13–3.62)	0.018	1.94 (1.053–3.558)	0.033
Triple-positive	2.08 (1.274–3.401)	0.003	1.51 (0.87–2.64)	0.145
Thrombocytopenia < 100 × 10^3^ μL	2.01 (0.99–4.07)	0.053	1.69 (0.80–3.59)	0.172
Hypertension	0.51 (0.32–0.83)	0.007	0.76 (0.42–1.38)	0.361
Diabetes mellitus	0.61 (0.32–1.18)	0.144	1.178 (0.54–2.60)	0.684
Dyslipidemia	0.63 (0.39–1.02)	0.062	0.91 (0.50–1.69)	0.770
Obesity	0.74 (0.45–1.23)	0.242	0.77 (0.43–1.35)	0.360

Adjusted model included age at first thrombosis, ANA positivity, triple aPL positivity, thrombocytopenia < 100,000/μL, hypertension, diabetes mellitus, dyslipidemia, and obesity. Model fit was acceptable (Hosmer–Lemeshow *p* = 0.706; Nagelkerke R^2^ = 0.110). Formal collinearity diagnostics did not demonstrate significant multicollinearity between predictors (maximum condition index 2.1).

**Table 4:** Multivariable logistic regression for predictors of arterial vs. venous first thrombotic event in APS.

Variable	Crude OR (95% CI)	*p*-Value	Adjusted OR (95% CI)	*p*-Value
Obstetric APS phenotype	0.99 (0.58–1.54)	0.817	1.25(0.72–2.18)	0.427
Age at first thrombosis (per year)	1.02 (1.00–1.04)	0.002	1.01 (0.99–1.03)	0.182
Severe thrombocytopenia (<100 × 10^3^/µL)	1.27 (0.66–2.39)	0.479	1.36 (0.64–2.93)	0.425
Diabetes mellitus	2.10 (1.12–3.93)	0.021	1.14 (0.54–2.93)	0.736
Hypertension	2.50 (1.54–4.08)	<0.001	2.61 (1.46–4.67)	<0.001
Dyslipidemia	1.80 (1.08–3.01)	0.024	1.64 (0.87–3.09)	0.129
Obesity	0.59 (0.35–0.99)	0.045	0.47 (0.26–0.85)	0.013
Lupus Anticoagulant positivity	0.79 (0.40–1.54)	0.438	0.85 (0.40–1.76)	0.648

Model fit statistics: Omnibus χ^2^(8) = 29.51, *p* < 0.001; Nagelkerke R^2^ = 0.143; −2 log likelihood = 326.36.

## Data Availability

The data presented in this study are available on request from the corresponding author due to privacy regulations and data-sharing restrictions related to multicenter electronic medical record–derived data.

## Supplementary Materials

The following supporting information can be downloaded at: https://www.mdpi.com/article/10.3390/diagnostics16121794/s1, Table S1. Missingness analysis comparing patients with available versus missing complement measurements (C3 and C4).

## Abbreviations

The following abbreviations are used in this manuscript:

APS	Antiphospholipid syndrome
tAPS	Thrombotic antiphospholipid syndrome
oAPS	Obstetric antiphospholipid syndrome
aPL	Antiphospholipid antibodies
LA	Lupus anticoagulant
aCL	Anticardiolipin antibodies
anti-β2GPI	Anti-β2 glycoprotein I antibodies
ANA	Antinuclear antibodies
HCQ	Hydroxychloroquine
LMWH	Low-molecular-weight heparin
OR	Odds ratio
CI	Confidence interval
IQR	Interquartile range
CKD	Chronic kidney disease
eGFR	Estimated glomerular filtration rate
