# Supplementary material for: Combined Obstetric and Thrombotic Complications Are Associated with Features Suggestive of an Immune-Enriched Phenotype Among Women with Thrombotic Antiphospholipid Syndrome (APS)
Source: Diagnostics (Basel). 2026 Jun 10;16(12):1794. doi: 10.3390/diagnostics16121794 (PMC13298550; doi:10.3390/diagnostics16121794)
Supplement: Supplementary file 1 [file diagnostics-16-01794-s001.zip › diagnostics-4292702-supplementary.pdf]

Supplementary Table S1

To evaluate potential selection bias related to incomplete complement testing, we compared baseline demographic, serologic, hematologic, thrombotic, and cardiometabolic characteristics between patients with available versus missing C3 and C4 measurements. Continuous variables are presented as mean ± standard deviation and categorical variables as number (%). Comparisons were performed using Student's t-test, chi-square test, or Fisher's exact test, as appropriate.

Supplementary Table S1. Missingness analysis comparing patients with available versus missing complement measurements (C3 and C4).

Variable	Available C3 (n=220)	Missing C3 (n=75)	p-value	Available C4 (n=207)	Missing C4 (n=88)	p-value
Age at first thrombosis, years	39.1 ± 16.6	43.3 ± 17.7	0.066	39.5 ± 16.9	42.2 ± 17.2	0.205
Triple-positive APS, n (%)	28 (14.9)	8 (11.1)	0.429	27 (15.4)	9 (10.6)	0.289
Lupus anticoagulant positivity, n (%)	186 (84.5)	63 (84.0)	0.910	175 (84.5)	74 (84.1)	0.922
aCL positivity (IgM/IgG ≥40 GPL/MPL), n (%)	52 (23.6)	12 (16.0)	0.166	49 (23.7)	15 (17.0)	0.206
Anti-β2 glycoprotein I positivity, n (%)	71 (37.8)	20 (27.8)	0.131	68 (38.9)	23 (27.1)	0.061
Thrombocytopenia <100 $\times 10^9$/L, n (%)	44 (20.0)	6 (8.1)	0.019	41 (19.8)	9 (10.3)	0.049
Hypertension, n (%)	92 (41.8)	37 (49.3)	0.257	85 (41.1)	44 (50.0)	0.157
Diabetes mellitus, n (%)	40 (18.6)	10 (13.7)	0.339	40 (19.5)	10 (12.0)	0.130
Dyslipidemia, n (%)	135 (61.4)	52 (70.3)	0.168	128 (61.8)	59 (67.8)	0.331
Obesity, n (%)	73 (34.6)	31 (44.3)	0.146	70 (34.7)	34 (43.0)	0.191

Abbreviations: APS, antiphospholipid syndrome; aCL, anticardiolipin antibodies; anti-β2GPI, anti-β2 glycoprotein I antibodies.
Complement measurements were performed according to routine clinical practice and were not systematically available for all patients.
